# OA14 Tocilizumab in steroid-refractory sight-threatening giant cell arteritis

**DOI:** 10.1093/rap/rkag113.014

**Published:** 2026-11-03

**Authors:** May Myat Mon, Eirini Giavri, Shadi Fallahranjbar

**Affiliations:** Internal Medicine, Milton Keynes University Hospital, Milton Keynes, United Kingdom; Rheumatology, Milton Keynes University Hospital, Milton Keynes, United Kingdom; Opthalmology, Milton Keynes University Hospital, Milton Keynes, United Kingdom

## Abstract

**Introduction:**

Bilateral visual involvement in giant cell arteritis (GCA) is uncommon, particularly after initiation of high-dose glucocorticoids (GC). Tocilizumab (TCZ), an interleukin-6 receptor inhibitor, is effective for inducing and maintaining remission, though evidence for visual recovery remains limited.

**Case description:**

A 90-year-old woman presented with acute right-eye visual loss following eight weeks of polymyalgic symptoms, headaches, scalp tenderness and tongue claudication in September 2025. She developed transient right amaurosis fugax three days prior to admission. Examination showed reduced right temporal artery pulsation and right optic disc swelling. Visual acuity (VA) was counting fingers in the right eye. Inflammatory markers were markedly elevated (ESR 89 mm/hr, CRP 42 mg/L), and temporal artery ultrasound demonstrated bilateral halo signs, confirming GCA.

Despite 3 days of intravenous methylprednisolone, she developed new left-eye visual symptoms with bilateral optic disc swelling consistent with arteritic anterior ischaemic optic neuropathy (AAION). Given progressive bilateral visual involvement while on GC, she was treated with intravenous TCZ (6 mg/kg), followed by oral prednisolone with taper and transition to weekly subcutaneous TCZ (162 mg).

Rapid subjective improvement occurred within 24 hours of TCZ administration, with stabilisation and partial recovery of VA. At ten weeks, VA in right eye improved to 6/47 and in left eye to 6/24 and inflammatory markers normalised. Optic disc examination showed slight right disc pallor and pink left disc with no evidence of disc swelling in both.

Follow-up: At ophthalmology review in January 2026, right-eye VA had markedly improved to 6/6 with pinhole, representing unexpected recovery, while left-eye VA remained stable at 6/15 with pinhole. Optic disc examination showed right disc pallor with superior retinal nerve fibre layer thinning and possible mild pallor on the left. By April 2026, she remained asymptomatic with stable unaided VA (right 6/12, left 6/15), flat optic discs, and no new ophthalmic concerns. She continued weekly TCZ with tapered oral prednisolone to 5mg once daily from June 2026 under rheumatology follow-up.

**Discussion:**

Total or partial visual loss is a serious complication of GCA, occurring in approximately 15–20% of cases, most commonly due to arteritic anterior ischaemic optic neuropathy (AAION). Bilateral involvement may develop in up to 25–50% of untreated patients, often within days to weeks, highlighting the narrow therapeutic window. Although high-dose glucocorticoids remain the cornerstone of treatment and are effective in preventing further visual loss in most cases, some patients continue to deteriorate despite therapy, particularly during the first week. Furthermore, GC have limited ability to reverse established ischaemic injury, making visual recovery uncommon, especially in those with complete visual loss.

Tocilizumab (TCZ), an interleukin-6 receptor inhibitor, is well established for inducing remission and reducing relapse in GCA, as demonstrated in the GiACTA trial. However, evidence regarding its role in acute visual manifestations remains limited. Emerging case reports suggest that TCZ may help stabilise vision in patients with progressive or steroid-refractory disease, although robust data demonstrating visual recovery are lacking.

In this case, progression of bilateral visual symptoms despite intravenous methylprednisolone indicated aggressive disease requiring early treatment escalation. The temporal association between TCZ initiation and subsequent visual stabilisation, followed by partial recovery, is notable, particularly given the degree of improvement in the right eye, which is rare in AAION. Although causality cannot be established, this observation supports consideration of earlier TCZ use in selected high-risk or refractory patients.

This case highlights that early diagnosis and guideline-concordant GC therapy do not completely eliminate the risk of ischaemic visual complications. Despite bilateral visual loss, the patient demonstrated significant visual improvement, particularly in the right eye, which was initially more severely affected. TCZ may represent a promising therapeutic option in such cases; however, further research is required to clarify its efficacy, optimal timing, and impact on visual outcomes in GCA.

**Key learning points:**

• Progressive ischaemic complications may still occur despite glucocorticoid therapy.

• Tocilizumab’s role as rescue treatment in sight-threatening GCA remains uncertain; improvement may reflect glucocorticoid effect or rapid IL-6 inhibition.

• Ischaemic complications may progress despite early, guideline-concordant glucocorticoid treatment, even within fast-track pathways.

• This case highlights the diagnostic and management challenges of acute, steroid-refractory, sight-threatening GCA.

• Tocilizumab may have a role as rescue therapy in selected cases, although causality and mechanisms underlying visual recovery remain uncertain.

• The case underscores the importance of rapid imaging, multidisciplinary decision-making, and individualised risk–benefit assessment when escalation of therapy is required.

• Monitoring disease activity and treatment response is challenging, particularly when inflammatory markers are suppressed by IL-6 inhibition.

